# Veno-venous ECMO for rapidly progressing interstitial lung disease: A multidisciplinary approach

**DOI:** 10.2478/jccm-2026-0006

**Published:** 2026-01-30

**Authors:** Samreen Tariq, Fiona O’Hannigan, Nizrull Nasir, Serena O’Brien, Brian Marsh, Jennifer Hastings, John Stack, Josephine Kelliher, Katherine O’Reilly

**Affiliations:** Mater Misericordiae University Hospital, Dublin, Ireland

**Keywords:** antisynthetase syndrome, rapidly progressing interstitial lung disease, veno-venous Extracorporeal Membrane oxygenation, myositis related interstitial lung disease, respiratory failure

## Abstract

**Introduction:**

This is a unique case of fulminant respiratory failure secondary to a rare cause of rapidly progressing ILD; antisynthetase syndrome (ASS). Failure to deliver timely multi-modal treatment in these cases can lead to increased morbidity and mortality.

**Case presentation:**

A previously healthy 27-year-old male presented to his local hospital with a 1-week history of malaise, shortness of breath and cough. Initial work up including bloods and imaging were suggestive of community acquired multi lobar pneumonia, for which he received treatment as per local guidelines. Unfortunately, despite broad empirical antimicrobial cover, he continued to deteriorate with worsening type-1 respiratory failure requiring intubation and subsequent institution of prone position ventilation. Extensive microbiological investigations yielded no positive results. On day 7 of admission immunological testing revealed an ENA screen positive for Jo-1 antibody and a diagnosis of ASS was made. Despite treatment with immunosuppression the patient’s condition rapidly deteriorated and the decision to support with V-V ECMO was made following MDT consideration as there remained uncertainty as to the extent of reversibility of the underlying condition.

**Conclusions:**

This patient recovered with combination of conventional immunosuppression, therapeutic plasma exchange and ECMO support. This case highlights Antisynthetase syndrome as a cause of reversible interstitial lung disease in the ICU and the importance of multi-disciplinary decision making and aggressive treatment approach in the management of such conditions.

## Introduction

Antisynthetase syndrome (ASS) is a rare autoimmune condition, characterised by the presence of autoantibodies against the aminoacyl-tRNA synthetase enzymes resulting in the development of one or more of the following entities-interstitial lung disease (ILD), myositis, arthritis. ILD associated with ASS is often rapidly progressive and failure to deliver timely multi-modal treatment can lead to ]increased morbidity and mortality [1].

The cornerstone of this treatment is systemic immunosuppression and this generally requires several weeks to become effective [2]. Veno-venous Extracorporeal Membrane oxygenation (ECMO) support can allow time for this immuno-modulatory treatment to work [3].

We hereby report a unique case with fulminant respiratory failure secondary to ASS, which recovered with combination of conventional immunosuppression, therapeutic plasma exchange and 37 days of ECMO support.

This case highlights ASS as a cause of reversible interstitial lung disease in the ICU and the importance of multi-disciplinary decision making and aggressive treatment approach in the management of such conditions.

## Case presentation

A previously healthy 27-year-old male presented to his local hospital with a 1-week history of malaise, shortness of breath and cough. Initial clinical and radiological examinations indicated multilobar consolidation and a diagnosis of community-acquired pneumonia was made and was treated according to local guidelines.

Despite broad empirical antimicrobial cover, he continued to deteriorate with worsening Type-1 respiratory failure requiring intubation and subsequent institution of prone position ventilation on day 5 of his admission as per PROSEVA trial protocol [4]. Lung protective ventilatory parameters were set as per ARDsnet protocol [5]. Neuromuscular blockade as per ACURAYS was administered [6]; all to maximize conventional medical therapy.

The patient was referred to our hospital for consideration of Veno-venous ECMO support on day 6 and was transferred conventionally to our facility using the Mobile Intensive Care Ambulance Service (MICAS). He initially responded to prone positioning with FiO_2_ requirements decreasing to 0.4 from 1.00 while supine.

**Fig. 1.**
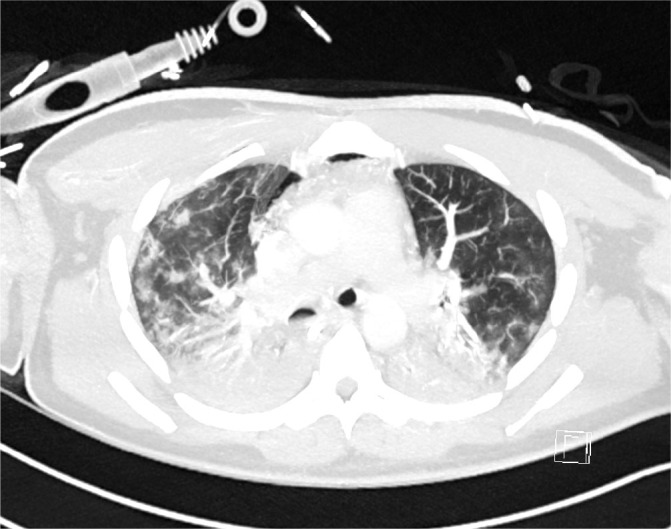
CT Thorax on Day 2 of admission showing extensive bilateral consolidation and surrounding nodular ground glass opacification, in keeping with multi-lobar pneumonia

Initial blood tests on transfer demonstrated raised inflammatory markers; CRP 193, White cell count of 20.3 with a predominant neutrophilia. Haemoglobin12.0g/L, platelet count 496×10^9^/L, creatinine 100umol/L, total protein 65g/L. ALT 112 IU/L, ALP IU/L, GGT 226 U/L, albumin 28g/L, total bilirubin 7umol/L. CK was unremarkable at 252 (ref range 44–272). A computed tomography (CT) thorax on transfer to our facility showed extensive bilateral consolidation and surrounding nodular ground glass opacification, in keeping with multi-lobar pneumonia (Figure 1). A bedside echocardiogram was essentially normal. Extensive microbiological investigations including sputum, blood, urine and broncho-alveolar lavage (BAL) culture for bacteria, mycobacteria and fungi, respiratory viral PCR from throat swab and BAL, legionella and pneumococcal urinary antigen yielded no positive results. There was no history of exposure to licensed medications or illicit drugs or factors known to cause lung disease. The presentation was one of severe Type 1 respiratory failure but notably there was no evidence of multi-system organ failure or hemodynamic compromise and hence was an unusual presentation for the initial working diagnosis of CAP with ARDS.

On day 12 of admission immunological testing revealed an ENA screen positive for Jo-1 antibody and myositis-specific autoantibodies were positive for Ro-52. Multispecialty input involving respiratory and rheumatology opinions were sought at that stage and a diagnosis of probable ASS was made based on provisional ACR/EULAR classification criteria 2024. A strongly positive Jo-1 antibody is quite specific for ASS in the appropriate clinical context (e.g. rapidly progressive ILD) [7].

Initial immunosuppression with intravenous (I.V.) methylprednisolone and I.V. cyclophosphamide was given the same day. Pre-biologic screen including hepatitis serology and Interferon Gamma-Release Array (IGRA) were sent prior to Rituximab being added. Due to a positive IGRA the patient was commenced on treatment for Latent TB infection with Isoniazid and pyridoxine (120 days treatment).

**Fig. 2.**
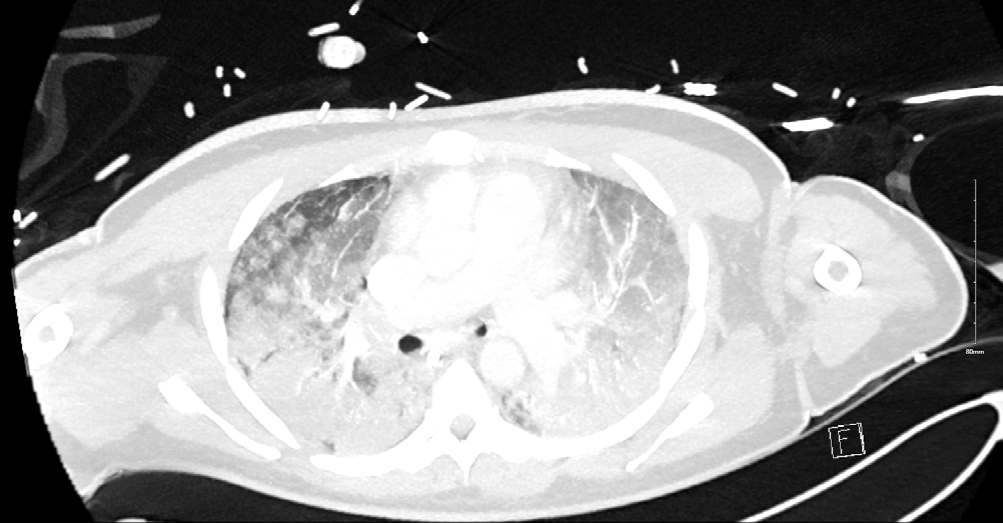
CT Thorax on Day 15 after clinical deterioration showing dense bilateral infiltrates throughout both lung in keeping with ARDS and possible organizing pneumonia. There was no pneumothorax or pleural effusions.

After receiving a course of methylprednisolone (1gm × 3), cyclophosphamide 500mg I.V. and Rituximab 1g the patient improved [2]. This was a transient effect (likely the effect of methylprednisolone) however and on day 15 of admission FiO_2_ requirements climbed to 1.00 and once again the PF ratio dropped to 9kpa. The patient was unresponsive to prone positioning on this occasion and with the employment of lung protective ventilatory protocols adequate ventilation was difficult to maintain. Given the significant deterioration a repeat CT scan was performed. This showed dense bilateral infiltrates which had significantly worsened since the first CT (Figure 2). The infiltrates were much more extensive throughout both lungs in keeping with ARDS and possible organizing pneumonia. MDT discussion took place, and a decision was made to support with VV ECMO as the pathophysiology was felt to be potentially reversible.

The patient was cannulated with a femoral 25 Fr60cm multistage access cannula to IVC and a 19Fr 18cm return cannula to right internal jugular. Flows 3.8L with sweep gas flow of 2.5. Percutaneous tracheostomy was performed on day 26 and he was mobilized with physiotherapy.

**Fig. 3.**
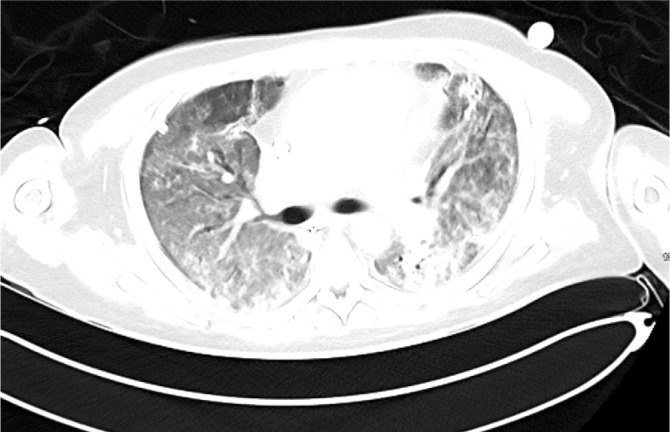
CT Thorax performed on Day 39 shortly after discontinuation of ECMO showed nodular and ground glass opacification which have improved significantly in the interim. There was some early traction bronchiectasis.

The patient received plasma exchange after consultation with the Haematology team, with a total of 5 sessions completed. While on ECMO he received another 1g rituximab and 2 further doses cyclophosphamide (500 mg I.V.). Gradually the patient improved. He tolerated 24 hours off sweep gas flow and was successfully decannulated from ECMO after 37 days. Ventilatory requirements immediately post ECMO decannulation showed acceptable peak pressures of 18 cmH_2_O with FiO_2_ 0.4 (PEEP 6 cmHO + Pressure support 10cm H_2_O). A CT was repeated on Day 39 shortly after ECMO was discontinued which showed bilateral improvement in both nodular and ground glass opacifications (Figure3). There was evidence of early traction bronchiectasis.

**Fig. 4.**
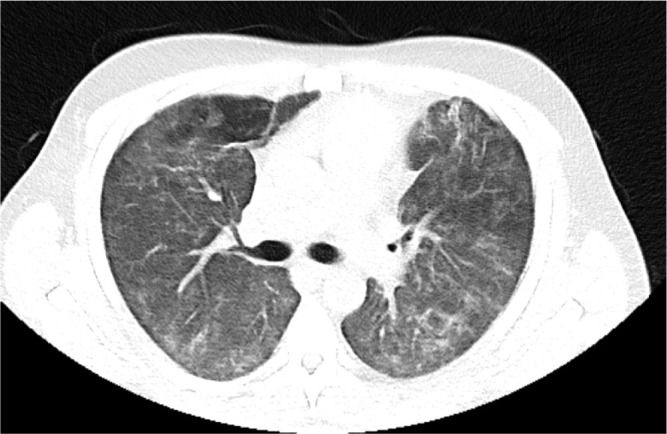
CT Thorax performed Day 92 prior to discharge home showing further improvement of both ground glass opacification and reticular shadowing with minor traction bronchiectasis.

**Fig. 5.**
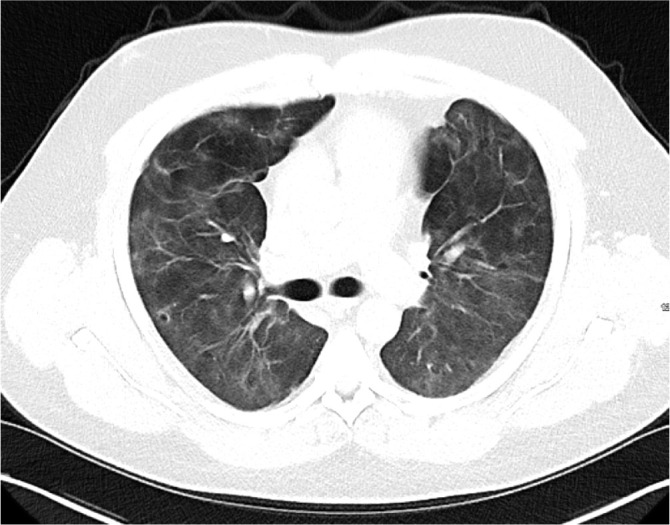
CT Thorax performed 2 years after initial presentation showing resolution of bilateral infiltrates. Ongoing upper lobe predominant reticulations and traction bronchiectasis were evident in keeping with interstitial fibrosis.

He was transferred to the ward for rehabilitation on day 68. A CT-thorax performed on Day 97 showed further improvement of bilateral infiltrates. There was residual ground glass opacification and reticular shadowing with minor traction bronchiectasis. These changes were consistent with interstitial fibrosis (Figure 4) and he was discharged home on 3L ambulatory oxygen, after 109 days. His hospital stay was complicated by reactivation of Cytomegalovirus and hospital acquired-pseudomonas pneumonia for which he was treated accordingly. He continues to have close follow-up with Rheumatology, Respiratory teams and attends the Critical Care follow up clinic. Currently he is weaning off the corticosteroid therapy and receives rituximab infusions every six months with mycophenolate mofetil 1g twice daily. Most recent CT Thorax performed 2 years post initial presentation showed resolution of bilateral infiltrates, however, upper lobe predominant reticulations and traction bronchiectasis were evident in keeping with interstitial fibrosis (Figure 5).

## Discussion

ASS first described by Marguerie and coworkers in 1990 is an autoimmune idiopathic inflammatory myopathy (IIM) [8]. It is characterized by ILD with the presence of autoantibodies against one of many aminoacyl transfer RNA (tRNA) synthetases.

Anti JO-1 is the most common antibody being detected in up to 70% patients with myositis and concomitant ILD [9]. Other anti-synthetase antibodies include anti-PL-7, anti-PL-12, anti-OJ, anti-EJ, anti-KS, anti-ZO, anti-YRA, and anti-Wa antibodies [1].

The diagnosis is based on the presence of the autoantibodies alongside detection of clinical signs of either ILD, inflammatory myopathy or polyarthritis affecting the small joints of hands [2]. Mechanic’s hands, which manifests as thickened and fissured skin at the tips of the fingers, is an important physical sign however it is present in only 30% patients with ASS [10].

Interestingly our patient did not have other clinical signs except for severe acute respiratory distress syndrome (ARDS) and high titer of anti JO-1 antibody on immune panel. It is appreciated however that a strongly positive Jo-1 antibody is usually quite specific for ASS in the appropriate clinical context (e.g. rapidly progressive ILD). Hence a high-weight is attached to presence of Jo-1 in the ACR/EULAR 2024 ASS classification criteria [7]. This presentation initially met criteria for probable ASS. Intriguingly after his discharge from hospital our patient developed myalgia and a raised serum CK level (consistent with inflammatory myositis). This met criteria for definite ASS. CT chest findings in ASS appear to predominate in the lower lobes [11].

The treatment approach to the ILD associated with ASS is not standardized and the evidence guiding management is mostly based on case series and reports [12]. Once the diagnosis is established, immunosuppression is the mainstay of the treatment. Glucocorticoids are the first line of treatment and other immunosuppressants like cyclophosphamide, azathioprine, tacrolimus, mycophenolate or rituximab should be initiated early in the disease course. For patients with rapidly progressing ILD(RP-ILD), the use of combination of Rituximab and Cyclophosphamide has shown to be superior to monotherapy in a growing number of case reports [13, 14].The American College of Rheumatology (ACR) most recently published guidelines for connective tissue disease associated ILD has conditionally recommended up-front combination therapy over monotherapy as first-line treatment for RP-ILD associated with CTD [15].

The effects of Plasma exchange (PLEX) on acute respiratory failure in ILD is not fully established.

In 2015, Omotoso published a report in which PLEX was found to be beneficial for the treatment of a patient with ASS associated ILD who was refractory to immunosuppression [16]. Bozkirli also reported a case of ASS with ILD who benefited from double-filtration plasmapheresis [17]. In 2021, Eller et al reported three Patients suffering from RP-ILD resulting from CTD-ILD, who benefited from and aggressive immunosuppression and extracorporeal blood purification [18]. Two thirds of the patients met the diagnostic criteria for ASS. In March 2023, Tekkatte et al, reported a case of ASS-ILD, treated with immunosuppression and PLEX with good clinical outcome [19]. In that case the ASS was related to ant PL-7 and Ro 52 and was treated with combination methylprednisolone and cyclophosphamide in addition to PLEX with the patient surviving to discharge home. Dequeker *et al*. have also reported TPE as a valuable adjunct therapy in acute exacerbation of CTD-ILD [20].

Determining the suitability of a patient for ECMO support can be complex. ECMO is not a treatment and despite improvement in the technology and devices there is still a considerable risk of complications including bleeding, infection, and long term physical and psychological trauma. Given the significant risks and potential complications associated with ECMO, its use in ASS must be carefully weighed against potential benefits. ECMO should only be considered when the anticipated therapeutic gain outweighs the associated risk.

Concerns in this case regarding suitability of ECMO support were the duration of ventilation prior to ECMO support, the patients relatively immunocompromised state and the uncertainty as to the extent of reversibility of the underlying condition. Reversibility is a key factor when determining whether ECMO support is appropriate, particularly where bridge to transplant is not possible.

Multi-disciplinary discussions with the respiratory and rheumatology teams who have experience in managing RP-ILD and an understanding of the pharmacology of the immunosuppressive agents were integral in this case. The advice from these specialist services was that the underlying pathophysiology was at least partially reversible. Prognosticating scores such as the widely used RESP score (Respiratory ECMO Prediction) were also useful to predict survival after ECMO for Severe Acute Respiratory Failure [21]. In this case the patient’s RESP score was 0 suggesting a 57% in-hospital survival rate.

The patient had been ventilated for 14 days at the point where VV ECMO suitability was initiated. Some studies have revealed poorer outcomes when patients are ventilated more than 10 days prior to cannulation [22]. Recent evidence suggests that duration of mechanical ventilation prior to ECMO cannulation does not have as big an influence on ICU mortality as previously thought. Hermann et al suggest that it may be the quality of ventilation rather than the quantity that is more important [23]. Overall, our patient had relatively protected ventilation for the duration of the 14 days prior to starting ECMO, hence the decision was made to initiate VV-ECMO while allowing the immunosuppression regimen take time to work.

Our case report adds to the growing body of literature on the role of VV ECMO in RP-ILD in patients diagnosed with ASS. Other case reports describing patients with ASS and RP-ILD successfully supported with VV ECMO are increasing, for example, Sampson et al, describe two patients with ASS who required VV ECMO. One was anti-Jo positive, the other anti-PL 12 positive. Both patients survived [24]. In addition, Chambrun et al describe the case of a 55-year-old man with ASS and RP-ILD who required VV-ECMO while being treated with steroids, tacrolimus, and tofacitinib. The patient survived to hospital discharge [25].

As ECMO availability increases, we believe that case reports on uncommon presentations are relevant. Myositis-associated RP-ILD is a heterogeneous entity that includes both the ASS and anti-MDA5 antibody subtypes.

## Conclusion

ASS and inflammatory interstitial lung disease has been seen to mimic community acquired pneumonia with ARDS [26]. Intensivists should consider a broad differential including ASS in cases of severe acute respiratory failure that are refractory to conventional treatment. RP-ILD is not a homogenous entity. Broader reviews of VV ECMO in myositis-associated RP-ILD suggest that VV ECMO may be justified in carefully selected patients, when the underlying disease is potentially reversible, early and aggressive immunosuppression is initiated, and suggest better outcomes with certain subtypes of disease [11]. ASS has been shown to carry a lower hospital mortality rate compared to dermato-pulmonary associated syndrome associated with anti MDA 5 antibodies [11] [27]. As we have demonstrated here, considerable reversibility is possible with aggressive multi-modality treatment. It is important to appreciate that these treatments take time - often several weeks to work optimally. There is no high-level evidence specifically supporting VV ECMO in anti-synthetase syndrome, however, case reports and small case series support its careful use in selected patients with acute, potentially reversible respiratory failure due to RP-ILD in the context of anti-synthetase syndrome.

Although this case report is a single patient experience, it adds to a growing volume of literature suggesting that VV ECMO may provide life sustaining support as a bridge to immunosuppressive therapy or decision-making in selected patients with respiratory failure related to ASS.
